# First record of the subfamily Oxytorinae (Insecta, Hymenoptera, Ichneumonidae) from the Oriental Region, with descriptions of two new species from Vietnam

**DOI:** 10.3897/BDJ.9.e69867

**Published:** 2021-08-13

**Authors:** Matthias Riedel, Lien Vu Van, Stefan Schmidt

**Affiliations:** 1 SNSB - Zoologische Staatssammlung München, Bad Fallingbostel, Germany SNSB - Zoologische Staatssammlung München Bad Fallingbostel Germany; 2 Vietnam National Museum of Nature, Vietnam Academy of Science and Technology, Hanoi, Vietnam Vietnam National Museum of Nature, Vietnam Academy of Science and Technology Hanoi Vietnam; 3 SNSB - Zoologische Staatssammlung München, Munich, Germany SNSB - Zoologische Staatssammlung München Munich Germany

**Keywords:** South-east Asia, biodiversity, new species, capacity building, Cuc Phuong National Park, Bach Ma National Park, biogeography

## Abstract

**Background:**

The Oxytorinae is a small subfamily of the family Ichneumonidae (Insecta, Hymenoptera) with the single genus *Oxytorus* Förster and 23 described species. Species were previously known to occur in the Palaearctic, Nearctic and Neotropical Regions.

**New information:**

The ichneumonid subfamily Oxytorinae is recorded for the first time from the Oriental Region. Two species, one from northern and one from central Vietnam, are described as new: *Oxytoruscarinatus* Riedel, sp. n. and *O.rufopropodealis* Riedel, sp. n.

## Introduction

The Oxytorinae is a small subfamily of Ichneumonidae (Hymenoptera) that includes only the genus *Oxytorus* Förster, with 23 described species found in the Palaearctic, Nearctic and Neotropic Regions (Broad et al. 2018). The biology and hosts of Oxytorinae are still unknown. The phylogenetic relationship of Oxytorinae within the Ichneumonidae is still a subject of research and Quicke et al. (2009) proposed an association with the polyphyletic subfamily Ctenopelmatinae. Recent results of a phylogenetic analysis agree with the placement of *Oxytorus* within Ophioniformes and most likely they are closely related to Ctenopelmatinae (Bennett et al. 2019). Ten species of *Oxytorus* Förster are known from the Eastern Palaearctic Region (Kasparyan et al. 2014, Sheng and Sun 2014), but this subfamily has not been found in the Oriental Region before. Here, we present our finding of two new species of *Oxytorus* Förster from Vietnam that were recently collected by the second author.

## Materials and methods

The present study resulted from capacity building training courses that were part of the ongoing German-Vietnamese VIETBIO project. The project aims at establishing the foundation for future biodiversity joint projects through capacity building and biodiversity research in Vietnam. Field courses took place in two national parks: Bach Ma National Park in central Vietnam (June 2018) and Cuc Phuong National Park in northern Vietnam (April-May 2019). The Bach Ma National Park demarcates the border between north and south Vietnam ranging from lowland to high mountains, with Bach Ma being considered as a biodiversity hotspot (Adler et al. 2016, Kunich 2003, Ngoc et al. 2016, Tobias 2014). The Cuc Phuong National Park in northern Vietnam is located at the junction between the temperate tropical and the subtropical regions. It is amongst the areas with the hightest documented diversity in Vietnam (Larsen et al. 2005, Eguchi et al. 2011). During the field courses, a range of different collecting methods were employed, including Malaise traps, Yellow Pan Traps and net sweeping.

Morphological terms and measurements follow Broad et al. (2018), except that the 1^st^ flagellomere was measured without the annellus. For the measurements, an Olympus SZX 7 stereomicroscope with dividing eyepiece was used. The figures were taken with an Olympus SC 30 CCD camera using the AnalySIS getIT software and processed using Helicon Focus (vers. 7.0) and, subsequently, enhanced with Adobe Lightroom Classic.

## Taxon treatments

### Oxytorus carinatus

Riedel sp. n.

990EEAD1-6039-5505-857F-40CA705EA397

EEDE1208-33B9-409A-AEBC-CFE62C061496

#### Materials

- **Type status:**Holotype. **Occurrence:** recordedBy: Stefan Schmidt; individualCount: 1; sex: female; lifeStage: adult; **Taxon:** scientificName: Oxytoruscarinatus; phylum: Arthropoda; class: Insecta; order: Hymenoptera; family: Ichneumonidae; genus: Oxytorus; specificEpithet: carinatus; taxonRank: species; scientificNameAuthorship: Riedel; **Location:** higherGeography: South-east Asia; country: Vietnam; countryCode: VN; stateProvince: Thừa Thiên-Huế; locality: Bach Ma National Park; verbatimElevation: 1236; decimalLatitude: 16.19368; decimalLongitude: 107.85596; geodeticDatum: WGS84; **Identification:** identifiedBy: Matthias Riedel; **Event:** eventID: ZSM-HYM-S271e; samplingProtocol: Yellow Pan Trap; eventDate: 09-06-2018; **Record Level:** type: PhysicalObject; language: en; institutionCode: VNMN; collectionCode: Entomology; basisOfRecord: PreservedSpecimen
- **Type status:**Paratype. **Occurrence:** recordedBy: Stefan Schmidt; individualCount: 1; sex: female; lifeStage: adult; **Taxon:** scientificName: Oxytoruscarinatus; phylum: Arthropoda; class: Insecta; order: Hymenoptera; family: Ichneumonidae; genus: Oxytorus; specificEpithet: carinatus; taxonRank: species; scientificNameAuthorship: Riedel; **Location:** higherGeography: South-east Asia; country: Vietnam; countryCode: VN; stateProvince: Thừa Thiên-Huế; locality: Bach Ma National Park, along trail to summit; verbatimElevation: 1294; decimalLatitude: 16.19488; decimalLongitude: 107.85365; geodeticDatum: WGS84; **Identification:** identifiedBy: Matthias Riedel; **Event:** eventID: ZSM-HYM-S2772; samplingProtocol: Yellow Pan Trap; eventDate: 10-06-2018; **Record Level:** type: PhysicalObject; language: en; institutionCode: ZSM; collectionCode: Entomology; basisOfRecord: PreservedSpecimen

#### Description

**Female holotype (Fig. 1)**

**Morphology.** Body length 8.0 mm (paratype 7.5 mm). Flagellum with 30 flagellomeres, long and filiform; 1^st^ flagellomere 4.3x as long as wide; 3^rd^ flagellomere 3.6x as long as wide; pre-apical flagellomere 1.25x as long as wide. Temple short, strongly and almost linearly narrowed behind eye, dorsally ca. 0.35x as long as eye (Fig. 1c). Distance of lateral ocellus to eye 1.0x ocellar diameter. Frons and vertex granulate, dull. Face finely punctate and granulate, dull. Clypeus smooth, with some fine setiferous punctures and basal transversal ridge. Malar space 0.5x as long as width of mandibular base. Mandible slender; upper tooth longer than lower one. Maxillary palp very long, 2^nd^ palpomere about as long as 3^rd^ hind tarsomere. Genal carina reaching hypostomal carina away from mandibular base; both carinae low.

Mesosoma covered with grey setae. Mesoscutum finely and densely punctate, shining. Notaulus impressed in frontal 0.5 of mesoscutum. Mesopleuron smooth, with fine, dense setiferous punctures ventrally and frontally, speculum smooth and shining (Fig. 1f). Metapleuron finely punctate; juxtacoxal carina absent. Scutellum moderately elevated, longer than wide, densely punctate, with complete lateral carina. Propodeum completely carinate, largely smooth and shining. Area basalis almost triangular, slightly longer than wide. Area superomedia with central transverse carina; frontal part almost triangular, caudal part rectangular and wider than long; anterior transverse carina (costula) reaching in its middle (Fig. 1b). Apical transverse carina of propodeum slightly lamelliform elevated at posterolateral edge of area dentipara. Hind femur 4.0-4.3x longer than wide; hind tibia 5.1-5.3x as long as wide, apical spurs slightly curved apically. Hind tarsus 1.55x as long as hind tibia. Claws simple, strongly curved, ca. 90° apically.

Areolet open distally (vein 3rs-m absent); vein 2m-cu slightly proximal to its middle. Vein 1cu-a slightly postfurcal. Nervellus of hind wing slightly inclivous.

Metasoma rather slender, mainly smooth and shining. 1^st^ tergite 3.0x longer than wide; postpetiole almost smooth, with fine dorso-lateral carina. 2^nd^ tergite 1.05x as wide as long, with distinct dorso-lateral longitudinal carina (Fig. 1e). Thyridium oval, situated at basal margin of 2^nd^ tergite. 2^nd^ and 3^rd^ tergites with fine dense setiferous punctures, shining. 2^nd^ and 3^rd^ sternites with median folds. Ovipositor sheath rather wide, pointed apically.

**Colour.** Black. Mandible, except teeth, apical 2/3 of clypeus, scape, pedicel and two basal flagellomeres reddish-yellow; following flagellomeres black; flagellomeres 13–19 with ivory stripes or rings. Palps, fore and mid-coxae, all trochanters and hind tarsus, except black base of hind metatarsus ivory. Tegula, spot on speculum, entire metapleuron and propodeum red. Metasoma black; 2^nd^ and 3^rd^ tergites with diffuse reddish spots apically; 2^nd^ and 3^rd^ sternites yellowish. Fore and mid-femora, tibiae and tarsi reddish-yellow; hind coxa red; hind femur red, narrowly infuscate at apex; hind tibia black, with reddish or yellowish external stripe. Wings hyaline; pterostigma brown.

#### Diagnosis

The species is characterised within the genus *Oxytorus* by the transverse carina of the area superomedia and the distinct lateral longitudinal carina of the 2^nd^ tergite. Due to the absent vein 3rs-m, the species runs to *O.luridator* (Gravenhorst, 1820) in the key to Palaearctic species (Kasparyan et al. 2014), but differs by its colour and the unique characters mentioned above.

#### Male

Unknown

#### Type depository

The holotype is deposited in the Vietnam National Museum of Nature, Vietnam Academy of Science and Technology, Hanoi, Vietnam and the paratype in the Zoologische Staatssammlung München, Munich, Germany.

### Oxytorus rufopropodealis

Riedel sp. n.

C5DC0F59-889C-5FA3-ADBA-6E213A01A669

0A513643-63C6-4FB0-AE27-56F21C310DF8

#### Materials

- **Type status:**Holotype. **Occurrence:** recordedBy: Stefan Schmidt, Olga Schmidt; individualCount: 1; sex: female; lifeStage: adult; **Taxon:** scientificName: Oxytorusrufopropodealis; phylum: Arthropoda; class: Insecta; order: Hymenoptera; family: Ichneumonidae; genus: Oxytorus; specificEpithet: rufopropodealis; taxonRank: species; scientificNameAuthorship: Riedel; **Location:** higherGeography: South-east Asia; country: Vietnam; countryCode: VN; stateProvince: Ninh Binh; locality: Cuc Phuong National Park, nr park center; verbatimElevation: 395; decimalLatitude: 20.34903; decimalLongitude: 105.59606; geodeticDatum: WGS85; **Identification:** identifiedBy: Matthias Riedel; **Event:** eventID: ZSM-HYM-S27e8; samplingProtocol: Yellow Pan Trap; eventDate: 05/07/2019; **Record Level:** type: PhysicalObject; language: en; institutionCode: VNMN; collectionCode: Entomology; basisOfRecord: PreservedSpecimen
- **Type status:**Paratype. **Occurrence:** recordedBy: Stefan Schmidt; individualCount: 1; sex: female; lifeStage: adult; **Taxon:** scientificName: Oxytorusrufopropodealis; phylum: Arthropoda; class: Insecta; order: Hymenoptera; family: Ichneumonidae; genus: Oxytorus; specificEpithet: rufopropodealis; taxonRank: species; scientificNameAuthorship: Riedel; **Location:** higherGeography: South-east Asia; country: Vietnam; countryCode: VN; stateProvince: Ninh Binh; locality: Cuc Phuong National Park, nr park center; verbatimElevation: 525; decimalLatitude: 20.35507; decimalLongitude: 105.60213; geodeticDatum: WGS85; **Identification:** identifiedBy: Matthias Riedel; **Event:** eventID: ZSM-HYM-S2774; samplingProtocol: Yellow Pan Trap; eventDate: 05/08/2019; **Record Level:** type: PhysicalObject; language: en; institutionCode: ZSM; collectionCode: Entomology; basisOfRecord: PreservedSpecimen

#### Description

**Female holotype (Fig. 2)**

**Morphology.** Body length 8.2 mm (paratype: 7.3 mm). Flagellum with 31 (paratype: 28) flagellomeres, long and filiform; 1^st^ flagellomere 4.2x as long as wide; 3^rd^ flagellomere 3.9x as long as wide; pre-apical flagellomere 1.1x as long as wide. Temple short, strongly and almost linearly narrowed behind eye, ca. 0.4x as wide as eye (Fig. 2c). Distance of lateral ocellus to eye 0.9x ocellar diameter. Frons and vertex granulate, dull. Face finely punctate and granulate, dull. Clypeus with some coarse setiferous punctures and basal transversal ridge. Malar space 0.6x as long as width of mandibular base. Mandible slender; upper tooth longer than lower one. Maxillary palps very long, 2^nd^ palpomere about as long as 3^rd^ hind tarsomere. Genal carina reaching hypostomal carina away from mandibular base; both carinae low.

Mesosoma covered with grey setae. Mesoscutum finely punctate and granulate, dull. Notaulus not impressed. Mesopleuron smooth, with band of fine longitudinal striation dorsally and rather dense setiferous punctures ventrally, speculum smooth and shining (Fig. 2e). Metapleuron punctate; juxtacoxal carina absent. Scutellum moderately elevated, longer than wide, punctate, with lateral carina in basal 0.2. Propodeum completely carinate, mainly and finely rugose, area petiolaris smoothened. Area basalis trapezoid, 1.5x longer than wide. Area superomedia almost rectangular, 1.3x as long as wide, apical carina weak or absent; anterior transverse carina (costula) reaching at its basal third (Fig. 2b). Apical transversal carina slightly lamelliform elevated at posterolateral edge of area dentipara. Hind femur 3.9x as long as wide; hind tibia 4.6x as long as wide, apical spurs curved apically. Hind tarsus 1.75x as long as hind tibia. Claws simple, strongly curved ca. 90° apically.

Areolet rhombic, pointed or shortly sessile frontally; vein 2m-cu slightly distal to its middle. Vein 1cu-a interstitial. Nervellus of hind wing slightly inclivous.

Metasoma rather slender, mainly smooth and shining. 1^st^ tergite 2.4x longer than wide; postpetiole with fine longitudinal striae, especially on lateral fields. 2^nd^ tergite 1.25x wider than long, without lateral carina. Thyridium oval, situated at basal margin of 2^nd^ tergite. 2^nd^ and 3^rd^ tergites with some fine setiferous punctures laterally. 2^nd^ and 3^rd^ sternites with median folds. Ovipositor sheath stout and wide.

**Colour.** Black. Scape, pedicel and two basal flagellomeres yellowish; following flagellomeres black; flagellomeres 10-17 with ivory stripes or rings. Palps, fore and mid-coxae, all trochanters and hind tarsus, except black base of hind metatarsus, ivory. Tegula, scutellum and postscutellum yellowish. Hind margin of mesopleuron, entire metapleuron and propodeum red. Metasoma black; petiolus partly reddish-brown dorsally; post-petiolus, 2^nd^ and 3^rd^ tergites with yellowish bands in apical halfs; 2^nd^ and 3^rd^ sternites yellowish. Fore and mid-femora, tibiae and tarsi pale yellowish; hind coxa reddish-yellow, with brown lateral stripe; hind femur red or chestnut-red; hind tibia blackish, with cream-yellow sub-basal stripe. Wings hyaline; pterostigma ochreous.

#### Diagnosis

The species runs to *O.kamikochianus* (Momoi, 1965) in the key of Kasparyan et al. (2014), but can been separated by the different colour of the propodeum and the metasoma.

#### Male

Unknown

#### Type depository

The holotype is deposited in the Vietnam National Museum of Nature, Vietnam Academy of Science and Technology, Hanoi, Vietnam and the paratype in the Zoologische Staatssammlung München, Munich, Germany.

## Supplementary Material

XML Treatment for Oxytorus carinatus

XML Treatment for Oxytorus rufopropodealis

## Figures and Tables

**Figure 1a.**
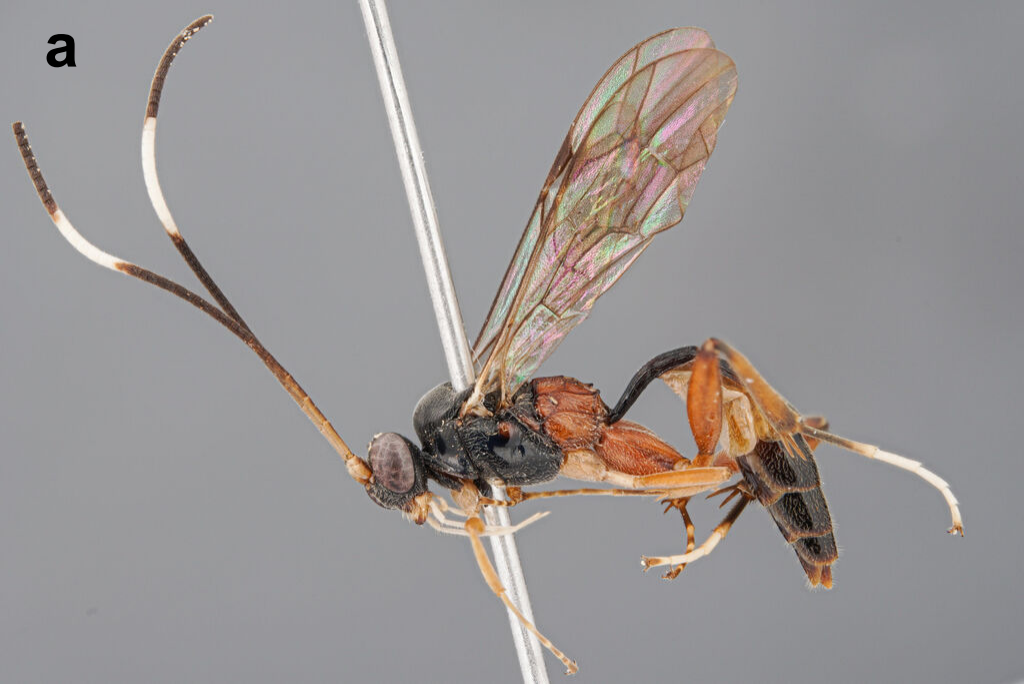
habitus lateral

**Figure 1b.**
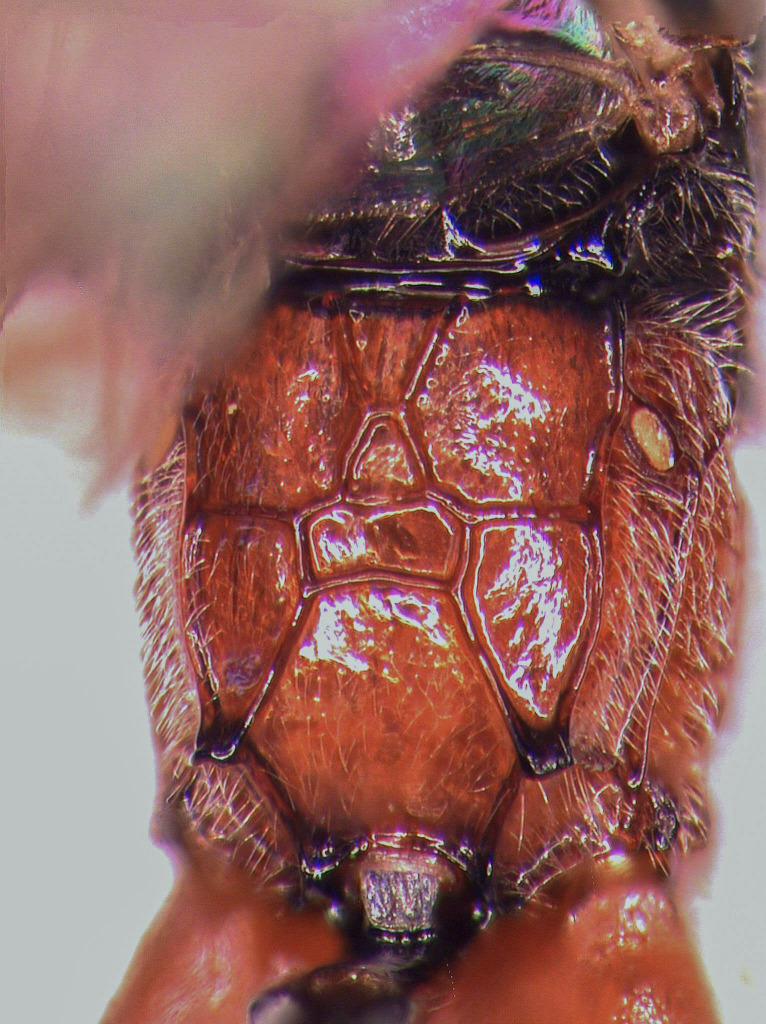
propodeum dorsal

**Figure 1c.**
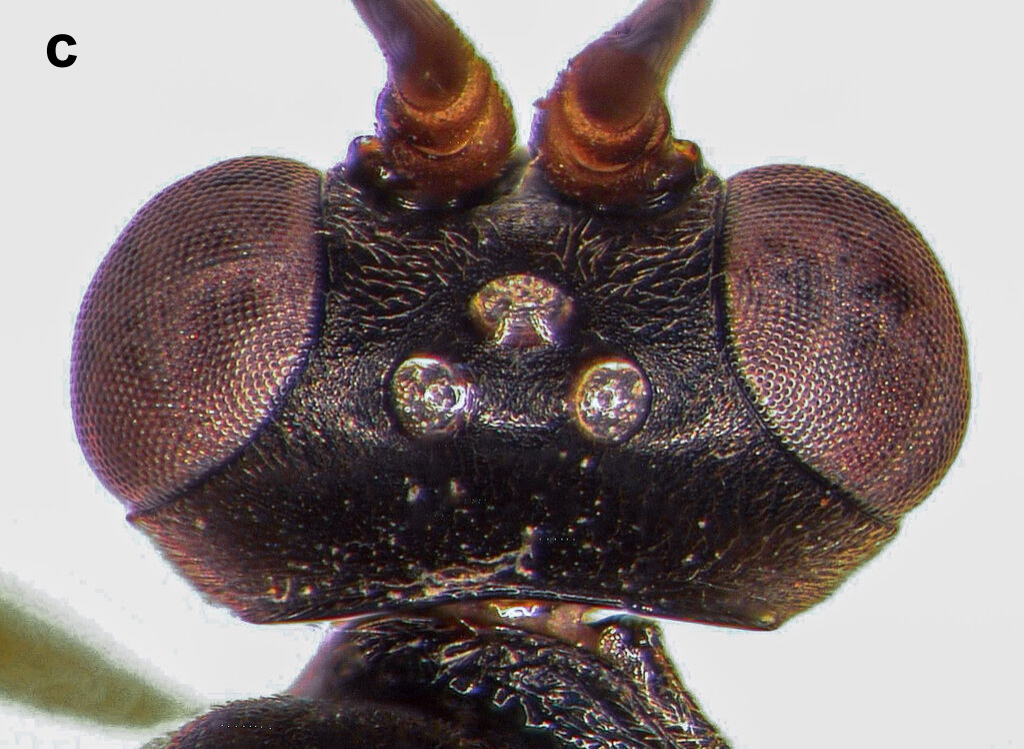
head dorsal

**Figure 1d.**
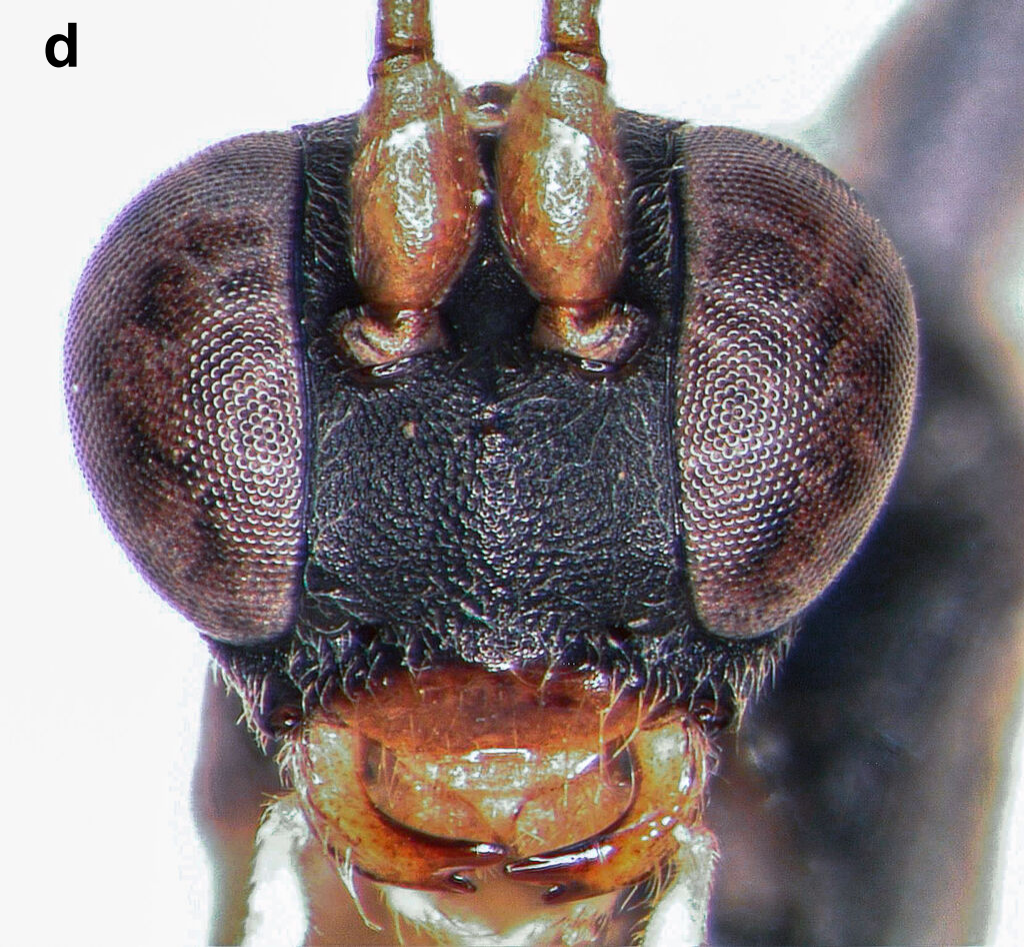
head frontal

**Figure 1e.**
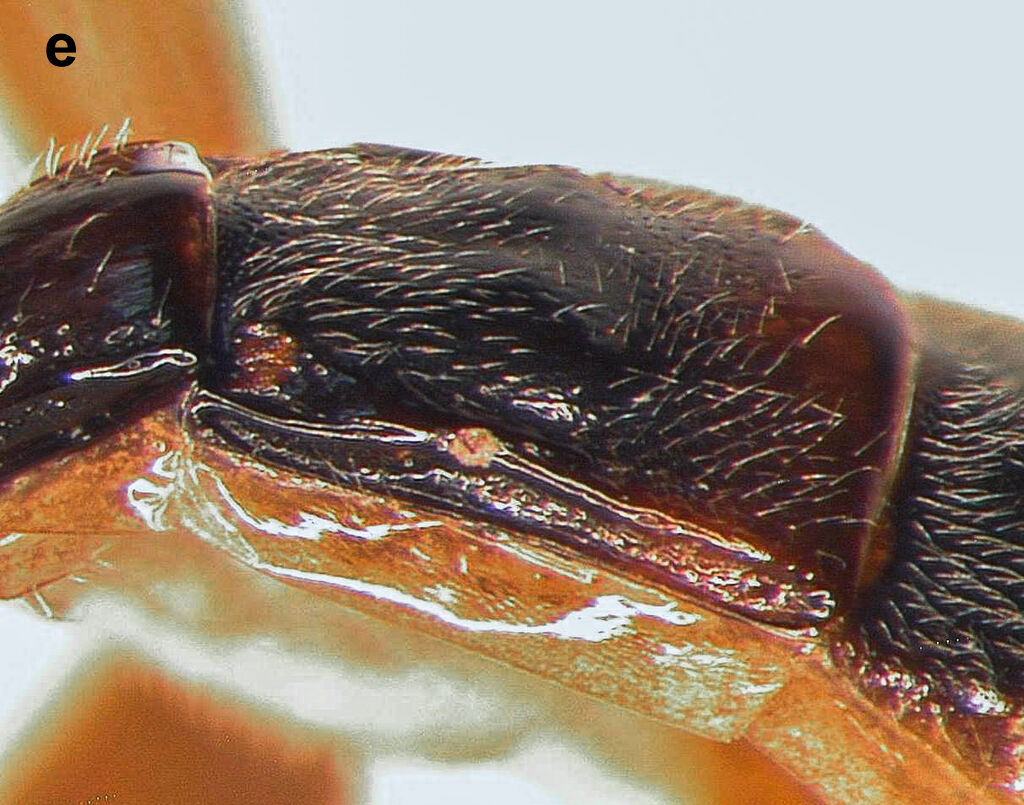
second tergite lateral

**Figure 1f.**
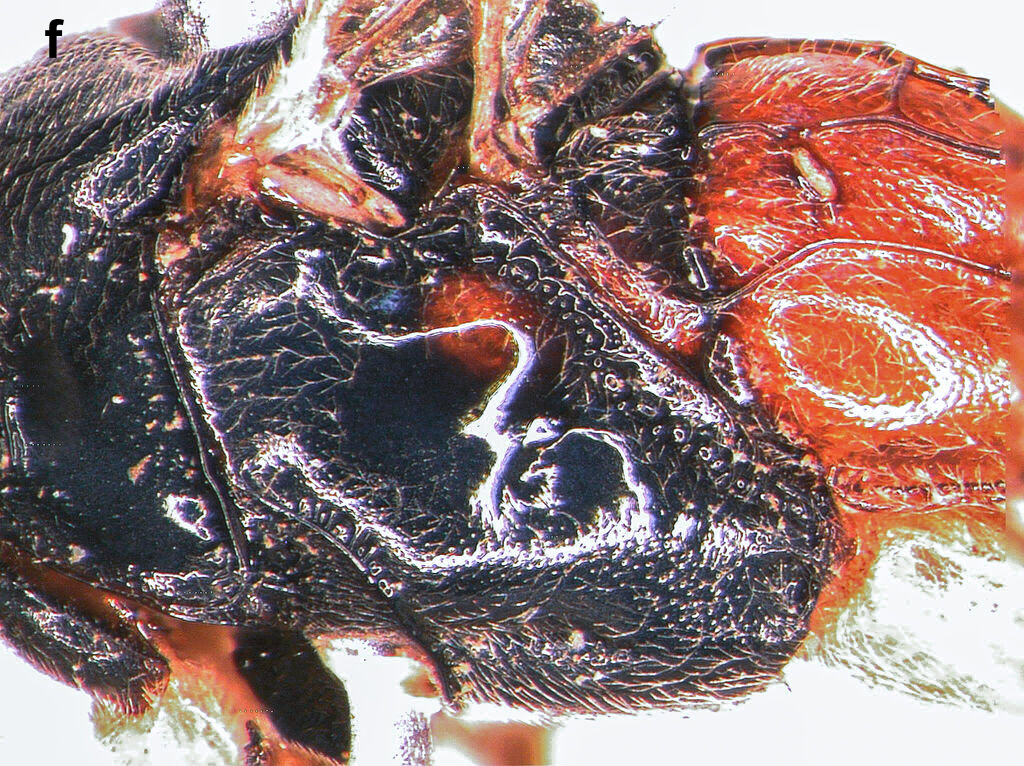
mesopleuron

**Figure 2a.**
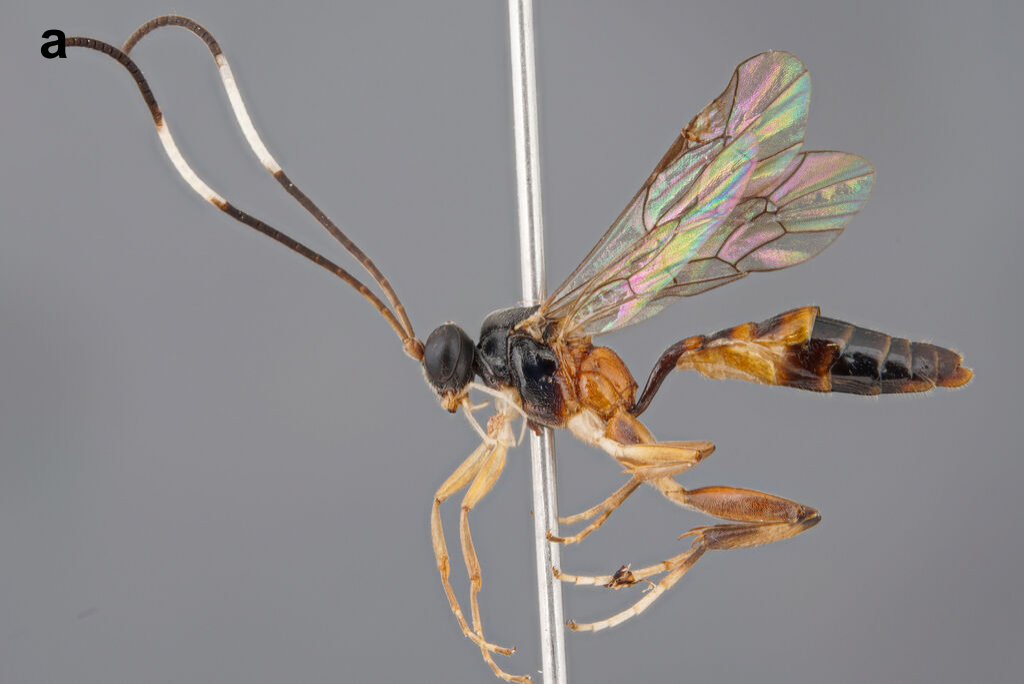
habitus lateral

**Figure 2b.**
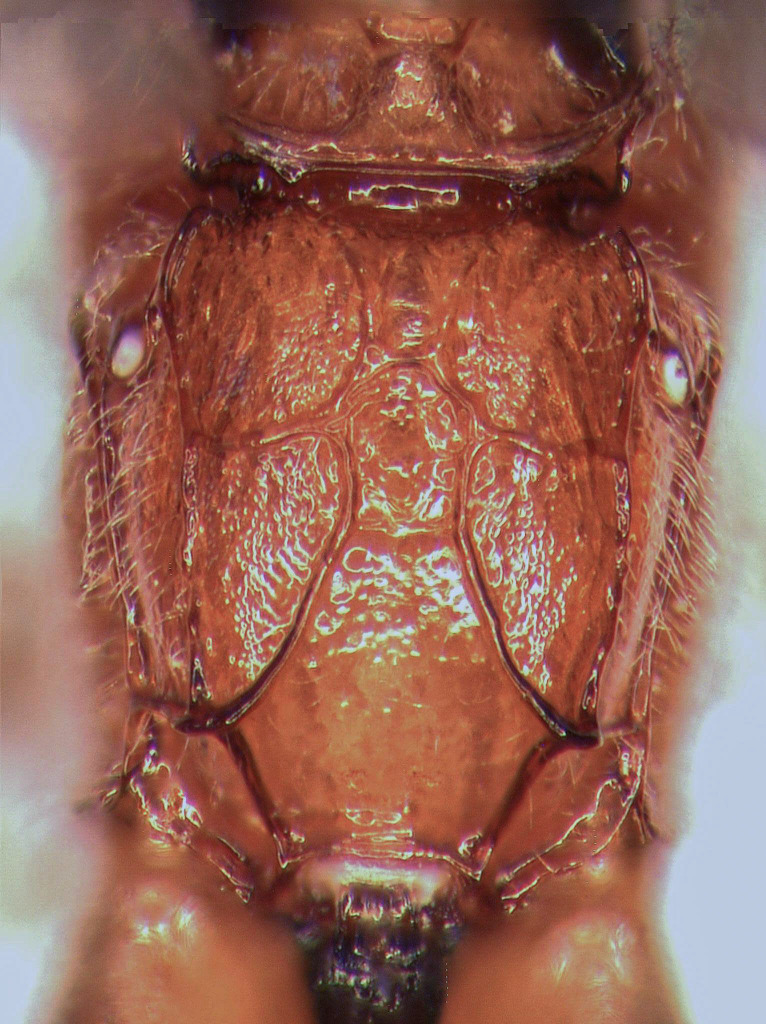
propodeum dorsal

**Figure 2c.**
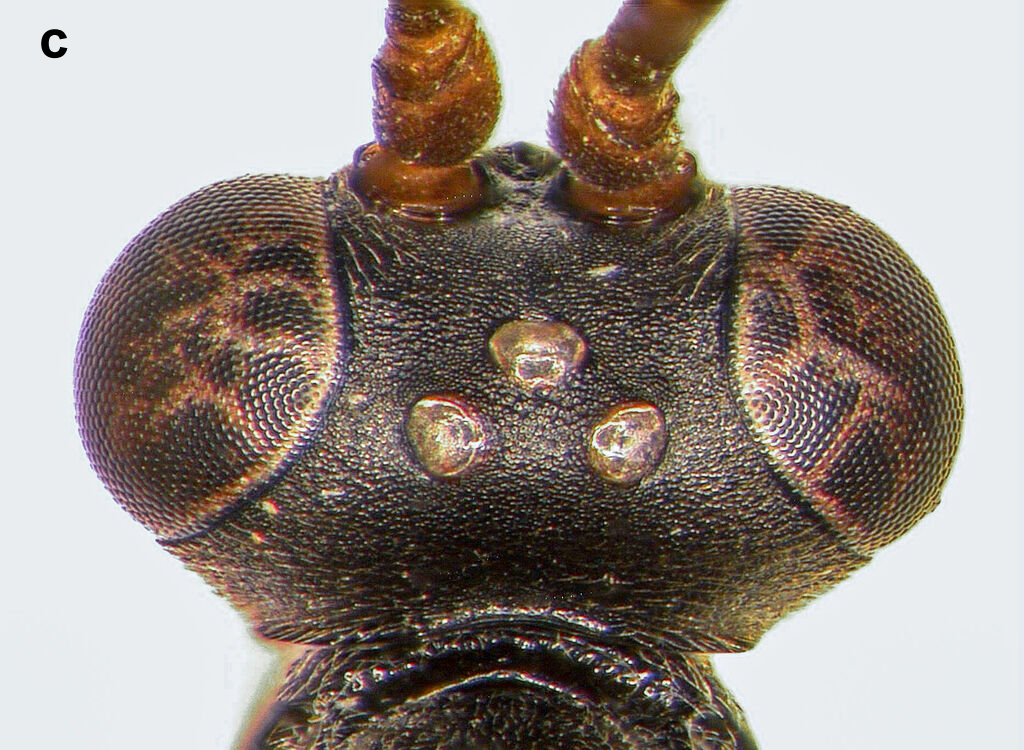
head dorsal

**Figure 2d.**
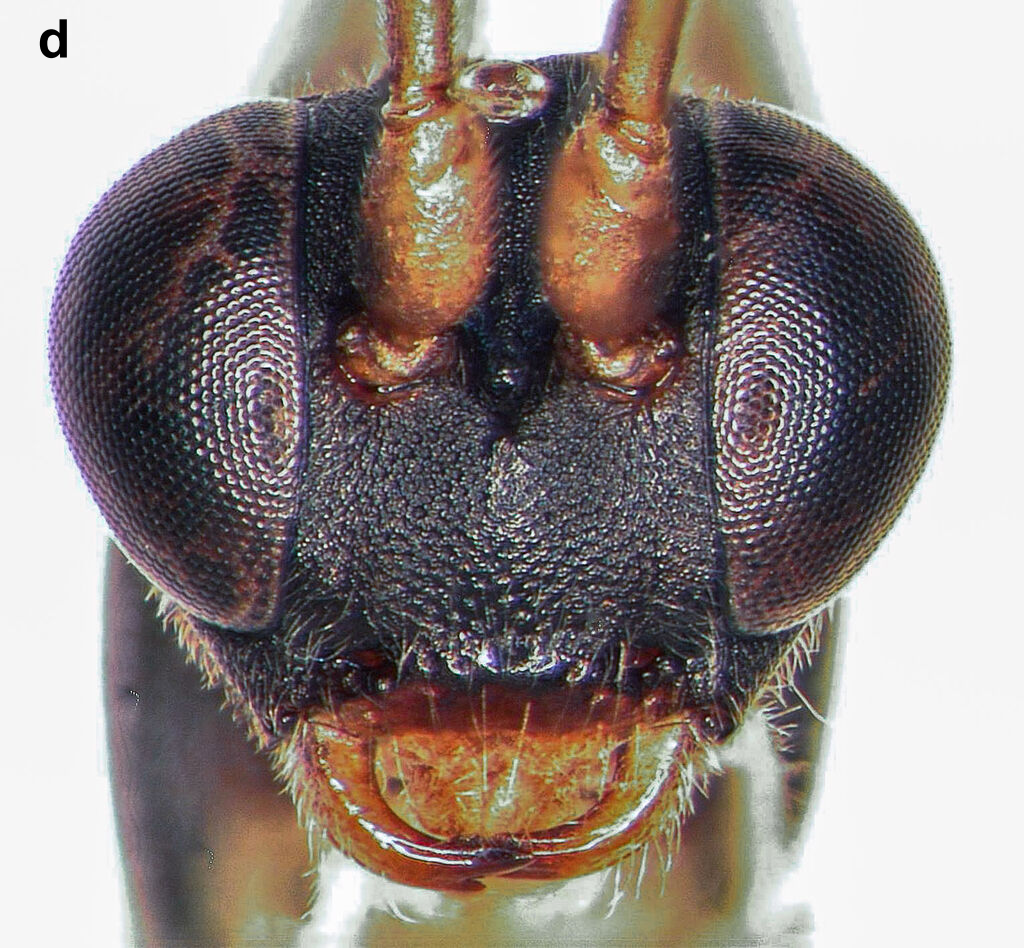
head frontal

**Figure 2e.**
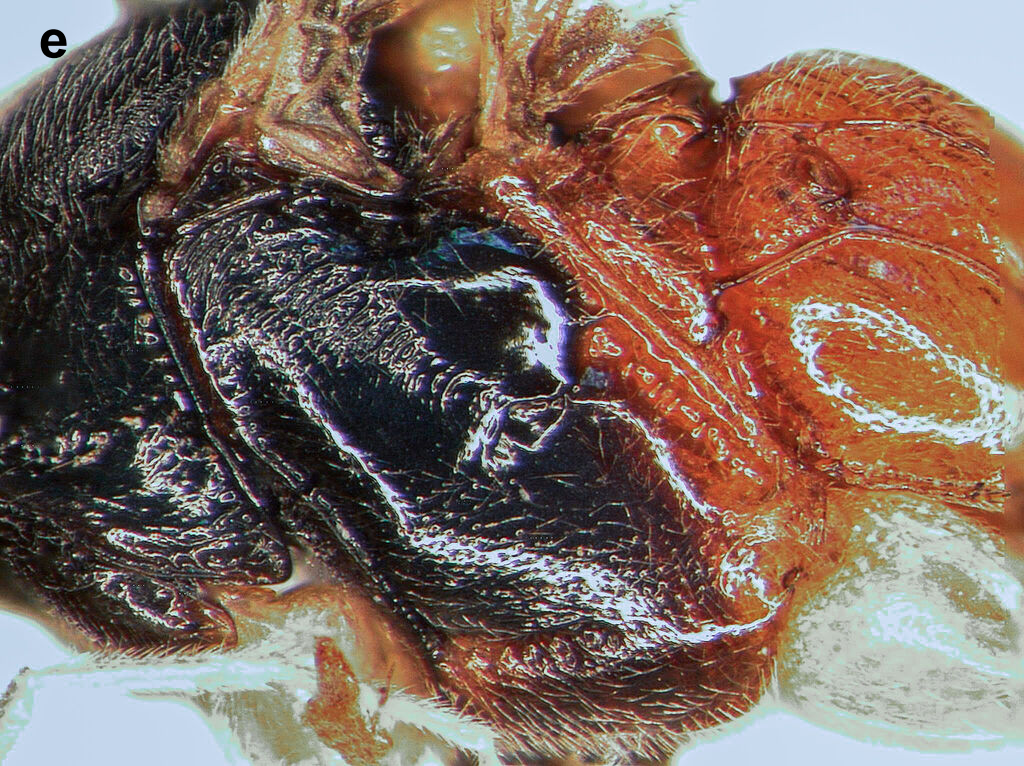
mesopleuron
